# Supplementary material for: Improving Neuropathological Reconstruction Fidelity via AI Slice Imputation
Source: Res Sq. 2026 Mar 31:rs.3.rs-9117500. Preprint. [Version 1] doi: 10.21203/rs.3.rs-9117500/v1 (PMC13060496; doi:10.21203/rs.3.rs-9117500/v1)
Supplement: 1 [file NIHPPRS9117500V1-supplement-1.pdf]

1 Supplement

1.1 Extended results of Surface reconstructions

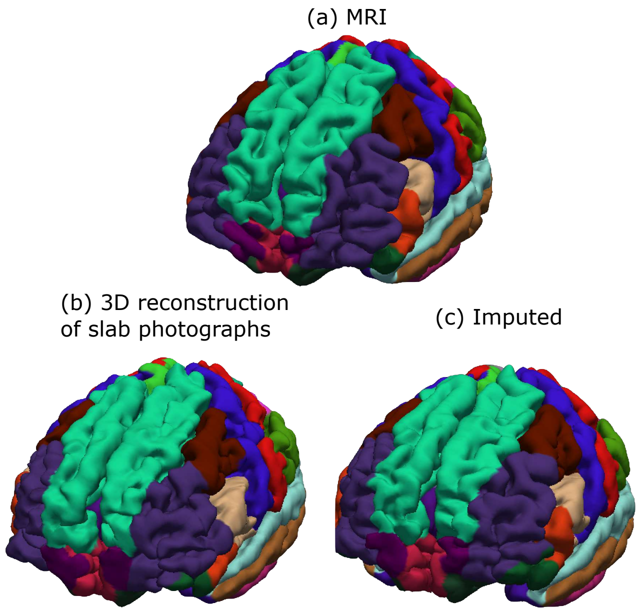

Fig. 1 Pial surfaces with overlaid parcellations, computed with Recon-Any on one case from the MADRC dataset. (a) Reference surface from MRI (gold standard). (b) Surface obtained from the 3D reconstruction of slab photographs. (c) Surface obtained with the proposed imputation.

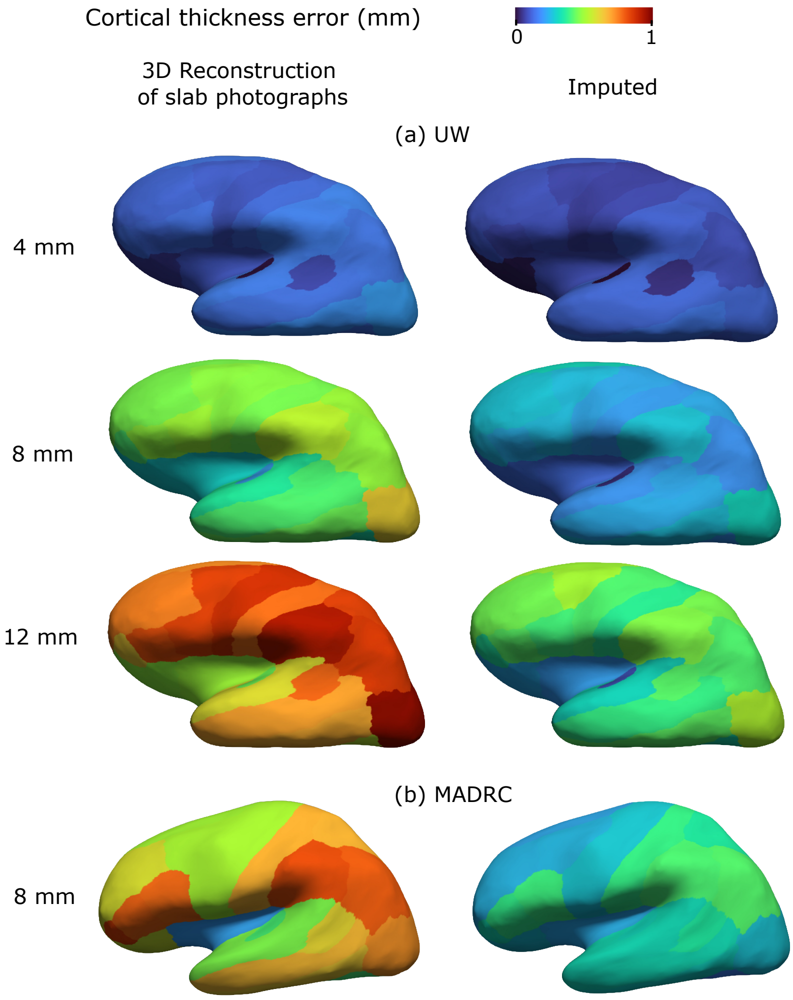

Fig. 2 Color coded illustration of cortical thickness error distributions of the original (left) and our proposed method of imputation (right), overlaid on inflated surface hemispheres computed with Recon-Any. (a) Distribution of errors from reconstructions of UW dataset at three slab thicknesses (4, 8, 12 mm) (b) Distribution of errors from MADRC reconstructions (8 mm).

Table 1 Surface and thickness errors (in mm) for Recon-Any of 3D photo reconstructions, computed against gold-standard MRI references. P-values from Wilcoxon Rank Sum statistical tests comparing both methods are reported for all evaluations.

**Pial Surface Error**				
**Dataset**	**UW – 4 mm**	**UW – 8 mm**	**UW – 12 mm**	**MADRC**
**Photo-recon**	0.845	1.084	1.313	1.767
**Imputed**	0.807	0.912	1.045	1.399
**p-Value**	<0.001	<0.001	<0.001	<0.001
**White Matter Surface Error**				
**Dataset**	**UW – 4 mm**	**UW – 8 mm**	**UW – 12 mm**	**MADRC**
**Photo-recon**	0.781	1.015	1.211	1.657
**Imputed**	0.763	0.854	0.969	1.279
**p-Value**	<0.001	<0.001	<0.001	<0.001
**Cortical Thickness Error**				
**Dataset**	**UW – 4 mm**	**UW – 8 mm**	**UW – 12 mm**	**MADRC**
**Photo-recon**	0.330	0.460	0.552	0.704
**Imputed**	0.261	0.276	0.331	0.367
**p-Value**	<0.001	<0.001	<0.001	<0.001

1.2 Extended results of Volume segmentations

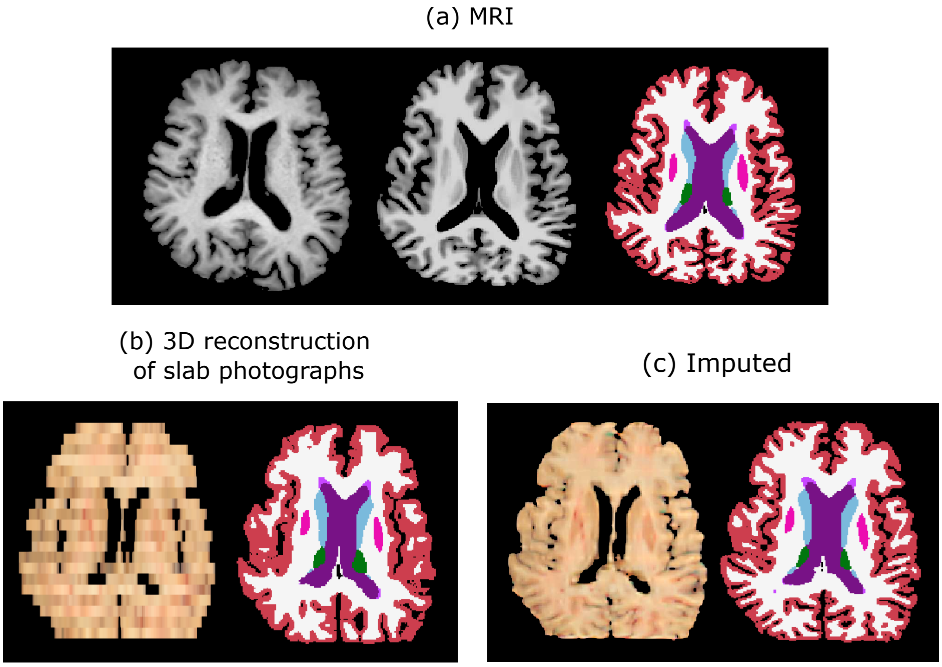

Fig. 3 Axial view of automated segmentations from one example of the MADRC dataset. (a) Native MRI prior to any pre-processing (left), gold-standard MRI processed with “SynthSR” (middle), and automated segmentation obtained with “SynthSeg” (right). (b) 3D Reconstruction of slab photographs (left) and automated segmentation with “Photo-SynthSeg” (right). (c) Imputed reconstruction (left) and automated segmentation with “SynthSeg” (right).

1.3 Extended results of Atlas registrations

Table 2 Region-specific Dice scores of automated segmentations of 3D reconstructions of photographs, before and after imputation. The gold standard segmentatations are obtained from MRI scans. The p-values are from Wilcoxon Rank tests (non-parametric, paired).

**MADRC**			
**Region**	**Photo-recon**	**Imputed**	**p-Value**
Amygdala	0.727	0.695	0.001
Caudate	0.689	0.690	0.837
Cortex	0.505	0.522	<0.001
Hippocampus	0.716	0.698	<0.001
Pallidum	0.712	0.716	0.554
Putamen	0.772	0.774	0.736
Thalamus	0.722	0.719	0.388
Ventricle	0.631	0.624	0.003
WM	0.670	0.678	<0.001
**UW – 4 mm**			
Amygdala	0.698	0.751	<0.001
Caudate	0.825	0.815	0.017
Cortex	0.770	0.778	<0.001
Hippocampus	0.710	0.732	0.003
Pallidum	0.714	0.793	<0.001
Putamen	0.797	0.845	<0.001
Thalamus	0.729	0.796	0.013
Ventricle	0.824	0.831	0.091
WM	0.825	0.850	<0.001
**UW – 8 mm**			
Amygdala	0.720	0.754	0.060
Caudate	0.825	0.827	0.023
Cortex	0.733	0.747	<0.001
Hippocampus	0.724	0.751	0.012
Pallidum	0.731	0.786	<0.001
Putamen	0.815	0.836	<0.001
Thalamus	0.767	0.832	0.035
Ventricle	0.831	0.838	0.336
WM	0.798	0.830	<0.001
**UW – 12 mm**			
Amygdala	0.722	0.739	0.033
Caudate	0.817	0.819	0.273
Cortex	0.707	0.712	0.001
Hippocampus	0.702	0.747	<0.001
Pallidum	0.694	0.799	<0.001
Putamen	0.802	0.837	<0.001
Thalamus	0.759	0.843	<0.001
Ventricle	0.820	0.829	0.028
WM	0.768	0.803	<0.001

Table 3 Region-specific Dice scores of (warped) atlas segmentations and gold-standard segmentations (from the MRIs). P-values computed with a (paired, non-parametric) Wilcoxon Rank Sum tests are also reported across datasets and slab thicknesses.

	**MADRC**		
**Region**	**Photo-recon**	**Imputed**	**p-Value**
Amygdala	0.409	0.503	0.005
Caudate	0.535	0.613	0.001
Cortex	0.449	0.493	<0.001
Hippocampus	0.480	0.574	<0.001
Pallidum	0.450	0.497	0.056
Putamen	0.574	0.646	0.001
Thalamus	0.555	0.624	0.002
Ventricle	0.545	0.581	<0.001
WM	0.629	0.656	<0.001
	**UW – 4 mm**		
Amygdala	0.539	0.605	<0.001
Caudate	0.752	0.735	0.099
Cortex	0.639	0.658	<0.001
Hippocampus	0.610	0.653	<0.001
Pallidum	0.604	0.625	0.001
Putamen	0.716	0.743	<0.001
Thalamus	0.742	0.757	0.001
Ventricle	0.796	0.778	<0.001
WM	0.764	0.769	<0.001
	**UW – 8 mm**		
Amygdala	0.485	0.592	<0.001
Caudate	0.726	0.749	<0.001
Cortex	0.602	0.643	<0.001
Hippocampus	0.573	0.626	<0.001
Pallidum	0.561	0.617	<0.001
Putamen	0.689	0.736	<0.001
Thalamus	0.709	0.751	<0.001
Ventricle	0.764	0.785	<0.001
WM	0.738	0.764	<0.001
	**UW – 12 mm**		
Amygdala	0.436	0.568	<0.001
Caudate	0.676	0.739	<0.001
Cortex	0.562	0.613	<0.001
Hippocampus	0.536	0.598	<0.001
Pallidum	0.446	0.550	<0.001
Putamen	0.632	0.710	<0.001
Thalamus	0.670	0.734	<0.001
Ventricle	0.720	0.782	<0.001
WM	0.707	0.745	<0.001
